# Optimization of a Navigation System for Autonomous Charging of Intelligent Vehicles Based on the Bidirectional A* Algorithm and YOLOv11n Model

**DOI:** 10.3390/s25154577

**Published:** 2025-07-24

**Authors:** Shengkun Liao, Lei Zhang, Yunli He, Junhui Zhang, Jinxu Sun

**Affiliations:** 1 Automotive and Transportation School, Tianjin University of Technology and Education, Tianjin 300222, China; liao_shengkun@163.com (S.L.); 18356854330@163.com (J.Z.); 15954868590@163.com (J.S.); 2 Department of Mechanical Engineering, Shandong Huayu University of Technology, Dezhou 253034, China; heyunli@163.com

**Keywords:** bidirectional A*, path planning, YOLOv11n, ROS system, corridor environment

## Abstract

Aiming to enable intelligent vehicles to achieve autonomous charging under low-battery conditions, this paper presents a navigation system for autonomous charging that integrates an improved bidirectional A* algorithm for path planning and an optimized YOLOv11n model for visual recognition. The system utilizes the improved bidirectional A* algorithm to generate collision-free paths from the starting point to the charging area, dynamically adjusting the heuristic function by combining node–target distance and search iterations to optimize bidirectional search weights, pruning expanded nodes via a greedy strategy and smoothing paths into cubic Bézier curves for practical vehicle motion. For precise localization of charging areas and piles, the YOLOv11n model is enhanced with a CAFMFusion mechanism to bridge semantic gaps between shallow and deep features, enabling effective local–global feature fusion and improving detection accuracy. Experimental evaluations in long corridors and complex indoor environments showed that the improved bidirectional A* algorithm outperforms the traditional improved A* algorithm in all metrics, particularly in that it reduces computation time significantly while maintaining robustness in symmetric/non-symmetric and dynamic/non-dynamic scenarios. The optimized YOLOv11n model achieves state-of-the-art precision (P) and mAP@0.5 compared to YOLOv5, YOLOv8n, and the baseline model, with a minor 0.9% recall (R) deficit compared to YOLOv5 but more balanced overall performance and superior capability for small-object detection. By fusing the two improved modules, the proposed system successfully realizes autonomous charging navigation, providing an efficient solution for energy management in intelligent vehicles in real-world environments.

## 1. Introduction

The rapid development and large-scale application of intelligent-vehicle technologies are driving the fundamental evolution of charging processes toward full automation and unmanned operation. In the context of developing an Autonomous Charging Navigation System (ACNS), the integration of an Automatic Charging System (ACS) and an Automatic Navigation System (ANS) has become a research hotspot [1,2,3]. Algorithms used for autonomous navigation and obstacle avoidance include Dijkstra’s algorithm [4], the A* algorithm [5], the APF algorithm [6], the RRT algorithm [7], etc. For navigation for autonomous charging in dynamic environments, the A* algorithm outperforms Dijkstra’s algorithm in computational speed, demonstrates greater stability than the artificial potential field (APF) algorithm, and offers distinct advantages in multi-sensor data fusion. However, the traditional A* algorithm still has inherent limitations. B.Y. [8] proposed a hybrid bidirectional path-search method that increases the cost of bidirectional path alignment. This method improves search speed but lacks dynamic constraints, leading to more difficult expansion and increased deviation of heuristic functions in complex environments. C.J. [9] developed the DCAE-BA* algorithm to constrain the expansion direction and minimize the number of expansion nodes of the bidirectional A* algorithm, reducing the number of explored nodes and optimizing path-planning length for diagonal target distances. However, in map environments with excessive numbers of turning points, smoothness of vehicle operation remains poor. J.C. [10] combined bidirectional A* with the dynamic window method to effectively handle obstacles in complex environments, but this approach increases path-planning length. There are multiple localization methods for intelligent vehicles in maps, including LiDAR localization, visual localization, millimeter-wave radar localization, multi-sensor fusion localization, and prior-map-based localization. In ACNS, navigation not only relies on algorithms but also requires the integration of cameras and radar systems for the recognition and localization of charging-pile areas and their corresponding charging piles. Y.G. [11] et al. addressed poor vehicle-positioning accuracy via SLAM-UWB synchronous positioning and recognition of visual information but failed to resolve the impact of color differences on SLAM; such a deficit may cause object-recognition errors. J. Terven et al. [12] conducted a detailed analysis of the evolutions and improvements from YOLOv1 to YOLOv8, showing that YOLOv8 achieves the best overall AP but suffers from model complexity and poor performance in small-object detection. Z. Chen [13] improved the accuracy and robustness of YOLOv11 in recognizing low-quality images by integrating an independent dehazing model (DEA-Net), though it exhibits weak performance in local refinement and global modeling in complex environments. S. Hu et al. [14] designed YOLOv11n with a hyperspectral image-denoising deep learning model (HCANet), optimizing feature fusion and attention mechanisms to enhance global–local feature interaction. However, it still demonstrates poor detection accuracy for small objects.

To sum up, the automatic navigation system for charging designed in this paper is faced with problems of poor path-planning optimality and path smoothness when using the bidirectional A* algorithm. While YOLOv11n is used for small-object recognition (e.g., charging piles), it suffers from insufficient performance in small-object detection and weak performance in environmental generalization. The improved algorithm employs a greedy approach to expand the search for the nearest points, redesigning the heuristic function by integrating the distance from the current node to the target and the number of search iterations. This aims to enhance the algorithm’s efficiency, minimize search time, and reduce path length. Additionally, it optimizes the trajectory using cubic Bézier curves to ensure a smoother driving experience. By incorporating the separable and self-attention mechanisms of HCANet into the YOLOv11n model, we enhance the adaptability of the DEA-Net model to complex environments, leading to the proposed CAFM. The improved YOLOv11n model effectively mitigates the impact of ambient brightness, enabling precise recognition of the characteristic information of small target objects.

## 2. Design Framework for an Autonomous Navigation System for Charging

### 2.1. Design of the Global Navigation Charging System

The Autonomous Charging Navigation System (ACNS) achieves full automation of intelligent-vehicle charging by integrating environmental perception, path planning, precise docking, and intelligent-control technologies. Without manual intervention, it leverages on-board sensors to autonomously avoid obstacles and navigate to a specified charging area based on the vehicle’s remaining battery level.

**Figure 1:**
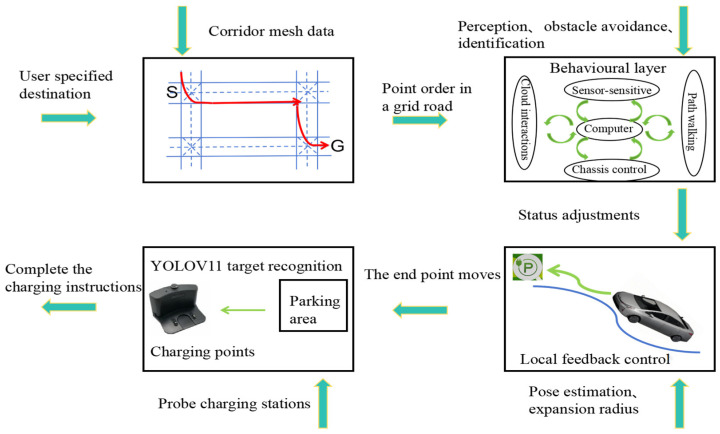
Framework for an autonomous navigation and charging system.

The ACNS shown in Figure 1 mainly consists of a path-planning function, a chassis-driving function, an autonomous-driving obstacle-avoidance function, and a charging-recognition and -planning function. The vehicle employs the improved bidirectional A* algorithm to plan paths, using radar and cameras to perceive, avoid, and identify obstacles and charging piles. The IMU sensor provides vehicle attitude information for positioning in environments with unstable GPS signals and supplies acceleration, angular velocity, and dynamic environmental perception data to support adjustments to chassis speed and steering. By fusion of lidar data, depth-camera data, and ordinary camera data, map information is established via the SLAM method [15], and the optimized bidirectional A* algorithm is used for trajectory planning, enabling the intelligent vehicle to dynamically avoid obstacles and reach the marked charging area. To ensure the intelligent vehicle accurately targets the specified charging area, YOLOv11n is used to recognize charging areas and piles, while infrared emitters and millimeter-wave radar prevent excessive collision between the vehicle and charging piles.

### 2.2. Infrared Navigation for Charging-Station Docking

**Figure 2:**
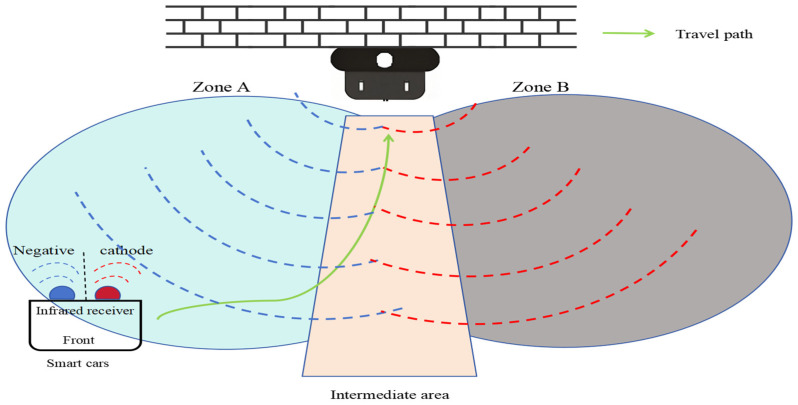
Charging pile and smart-vehicle signal docking.

The docking between the charging pile and the intelligent vehicle is illustrated in Figure 2. The infrared emitter of the charging pile emits signals in Areas A and B. When the intelligent vehicle receives signals from Area A or B, it first moves to the Intermediate Area, then slowly reverses to the positive/negative electrode modules of the charging pile. The ECU (Electronic Control Unit) detects whether the current and voltage signals are docked; if not, it recalibrates the position of the intelligent vehicle.

## 3. Improvement of the Bidirectional A* Algorithm

### 3.1. Improvement of the Bidirectional A* Algorithm for Path Planning Using a Greedy Approach

The traditional bidirectional A* algorithm performs searches from the start and end points simultaneously. When the search regions of the two directions “meet,” the paths are merged to obtain the optimal solution, which theoretically reduces the search space and improves efficiency [16,17]. However, as the number of nodes expands, the computational workload increases exponentially, leading to prolonged calculation time and failure to guarantee planning of the shortest path. To address the issue of ensuring the shortest local path in path planning, a greedy optimization algorithm [18] is employed for exploration. This algorithm evaluates the direct cost of moving from the current node to the target and repeatedly selects the node closest to the target for expansion at each step. This is shown in Equation (1), as follows:

(1) $$h\left(n\right)=\sqrt{{\left(x_{g}-x_{n}\right)}^{2}+{\left(y_{g}-y_{n}\right)}^{2}}$$

For the expansion node n of h(n), selecting the node with the minimum path as the next expansion node for path planning can ensure that the path between nodes is the shortest available.

The global path search continues to adopt the bidirectional A* approach, with bidirectional searches starting from both the target point and the starting point. This approach is shown in Equation (2), as follows:

(2) $$f_{m}(n)=g_{m}(n)+h_{m}(n)>f_{best}(n)$$

where $g_{m}(n)$ is the forward search and $h_{m}(n)$ is the backward search.

With the addition of an increased forward dynamic weight $W_{S}$ and an increased backward dynamic weight $W_{G}$, one obtains Equations (3) and (4), as follows:

(3) $$f_{1}(n)=g_{1}(n)+W_{S}h_{1}(n)$$

(4) $$f_{2}(n)=g_{2}(n)+W_{G}h_{2}(n)$$

where g(n) is the actual cost of moving from the starting point to the current node, h(n) is the estimated cost of moving from the current point to the target point, and f(n) is the cumulative cost of moving from the starting point to node n.

Linear adjustments are based on the current node. This is shown in Equations (5) and (6), as follows:

(5) $$w_{S}=w_{min}+\frac{d(n,G)}{D_{max}}(w_{max}-w_{min})$$

(6) $$w_{G}=w_{min}+\frac{d(m,S)}{D_{max}}(w_{max}-w_{min})$$

where d(n,G) and d(n,S) are the distances between the forward and backward current search nodes and the target point, respectively; $D_{max}$ is the maximum distance; and w is the weight.

An exponential adjustment of the search iteration count is applied as in Equations (7) and (8), as follows:

(7) $$w_{S}=w_{0}\cdot \alpha^{\frac{i_{S}}{I_{max}}}$$

(8) $$w_{S}=w_{0}\cdot \alpha^{\frac{i_{G}}{I_{max}}}$$

where $i_{S}$ is the number of forward search iterations, $i_{G}$ is the number of backward search iterations, $I_{max}$ is the maximum number of iterations, $w_{0}$ is the initial weight, and α is the decay factor (0 < α < 1).

The approach also takes into comprehensive consideration the distance between the current node and the target, as well as the number of search iterations. This is shown in Equations (9) and (10), as follows:

(9) $$w_{S}=w_{0}\cdot (\beta_{1}\cdot \frac{d(n,G)}{D_{max}}+\beta_{2}\cdot (1-\frac{i_{S}}{I_{max}}))$$

(10) $$w_{G}=w_{0}\cdot (\beta_{1}\cdot \frac{d(n,S)}{D_{max}}+\beta_{2}\cdot (1-\frac{i_{G}}{I_{max}}))$$

where $\beta_{1}$ is the distance weight coefficient, β2 is the iteration-number weight coefficient, and β1 + β2 = 1.

The weight is adjusted more flexibly by this formula through a weighted combination of the influences of distance and the number of iterations, and efficiency and optimality are balanced in different stages of the search.

### 3.2. Optimization of Redundant Node-Pruning Strategy for Bidirectional A* Algorithm and Cubic Bézier Curves

**Figure 3:**
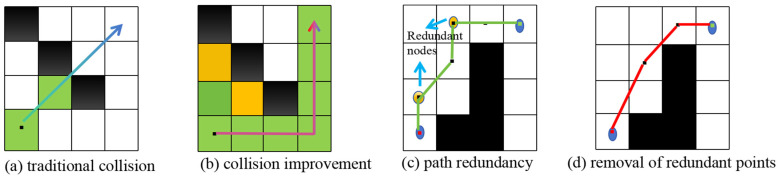
Effect diagram representing the redundant node-pruning strategy.

In a grid map, without the constraint of the inflation area, the phenomenon shown in Figure 3—a will occur, where the path directly passes through the gaps between obstacles. Therefore, constraints are imposed on the expansion of new nodes during octagonal exploration. This is shown in Equation (11), as follows:

(11) Newnode(x+1,y)&&Newnode(x,y+1)=0

If octagonal judgment shows no connected obstacles blocking expansion, the effect is as shown in Figure 3b. However, traditional A* and bidirectional A* both prioritize minimizing path length. Now, set three adjacent random nodes in the explored expanded nodes as P1, P2, and P3. If the three points are collinear, the middle point can be removed. By traversal of all node sequences, the path in Figure 3c can be optimized to that in Figure 3d.

The start and end points are first inserted into the open1 and open2 lists, respectively. Four adjacent expanded nodes are then backtracked and denoted as a, b, c, and d. Following the traversal of these nodes, a smooth curve is generated through cubic Bézier curve optimization [19]. Interpolation is employed only when optimizing paths with Bézier curves, while discrete nodes are maintained in other scenarios. This is shown in Equation (12), as follows:

(12) $$B(t)=(1-t{)}^{3}P_{0}+3t(1-t{)}^{2}P_{1}+3t^{2}(1-t)P_{2}+t^{3}P_{3},t\in [0,1]$$

Path length is calculated by accumulating distances between discrete points, and path smoothness is measured as the reciprocal of the standard deviation of angles between adjacent vectors. This is shown in Equations (13) and (14), as follows:

(13) $$S=\frac{1}{std\left(\left\{\theta_{i}\right\}\right)+\epsilon }$$

(14) $$\theta_{i}=arccos(\frac{{\overset{\to }{v}}_{i}\cdot {\overset{\to }{v}}_{i+1}}{∥{\overset{\to }{v}}_{i}∥\cdot ∥{\overset{\to }{v}}_{i+1}∥})$$

where ${\overset{\to }{v}}_{i}$ is the vector between adjacent points $P_{i}$ and $P_{i-1}$, $\theta_{i}$ is the angle between two vectors, γ is 0.001 (to avoid division-by-zero errors when the standard deviation is zero), and S is the smoothness ratio (where a greater value indicates a smoother path).

### 3.3. Testing and Analysis of MATLAB (2022-a) Algorithms

**Figure 4:**
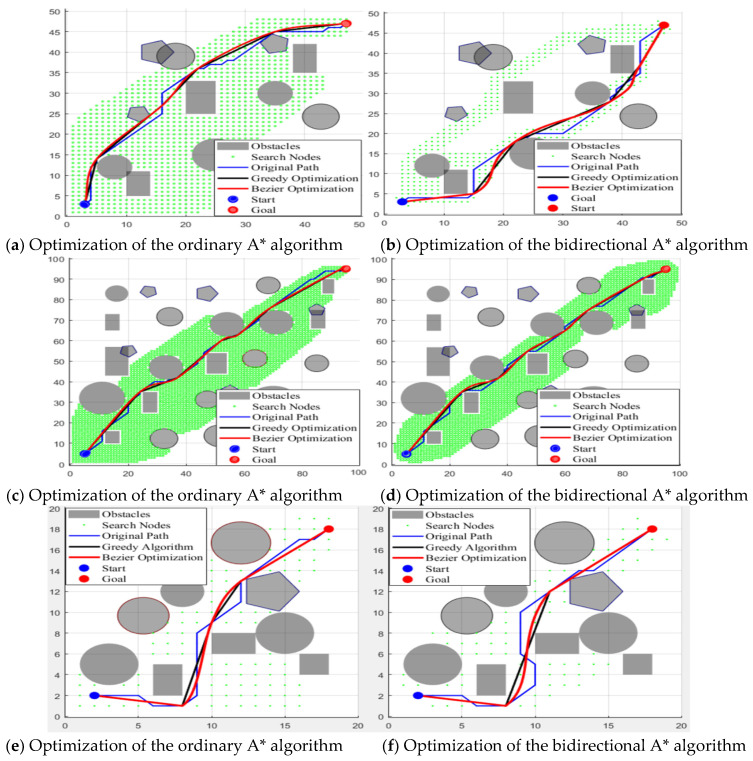
Graphs showing comparison of optimization performance between two algorithms.

This experiment assesses the path-planning performance of the ordinary A* algorithm and the bidirectional A* algorithm across maps of varying dimensions (20 × 20, 50 × 50, and 100 × 100). Initially, greedy optimization is applied to the original paths; this approach is followed by Bézier curve optimization with interpolation on the greedily optimized paths. The evaluation encompasses key metrics such as planning time, the number of expanded nodes, path length, and path smoothness. Corresponding experimental results are visualized in Figure 4 and tabulated in Table 1, providing a comprehensive comparison of algorithmic performance given different map sizes and optimization techniques.

This paper conducts a comparative study on the path-planning performance of traditional A* and bidirectional A* algorithms under 20 × 20, 50 × 50, and 100 × 100 grids, combined with experimental analyses using greedy optimization and cubic Bézier curve-optimization strategies. Results show that bidirectional A* demonstrates significant advantages in planning time (4.9 × faster than traditional A* on 100 × 100 maps) and number of searched nodes (43.6% reduction on 100 × 100 maps), with efficiency improvements becoming more pronounced as grid density increases; these results verify the efficiency of bidirectional search in complex environments. Greedy optimization significantly shortens paths for bidirectional A* (5.2% reduction compared to original paths on 100 × 100 maps), outperforming traditional A* optimization (5.0% reduction). As indicated by the data in Table 1 and Figure 4, although Bézier curve optimization after greedy processing sacrifices some advantages in path length, it enhances curve smoothness, with better optimization effects for bidirectional A* than for traditional A*. The developed “efficiency–distance–smoothness” trade-off solution is validated via visualization, confirming that the optimized bidirectional A* algorithm excels in efficiency and path-quality optimization for complex environments, adapting to the requirements of robotic navigation.

## 4. Visual Recognition and Localization of Charging Areas and Ports Based on the Optimized YOLOv11n Model

When the vehicle approaches the charging area, the image information collected by the camera is processed by the optimized YOLOv11n model to achieve high-precision visual recognition and localization of the charging area and charging port.

### 4.1. YOLOv11n Algorithm Framework

**Figure 5:**
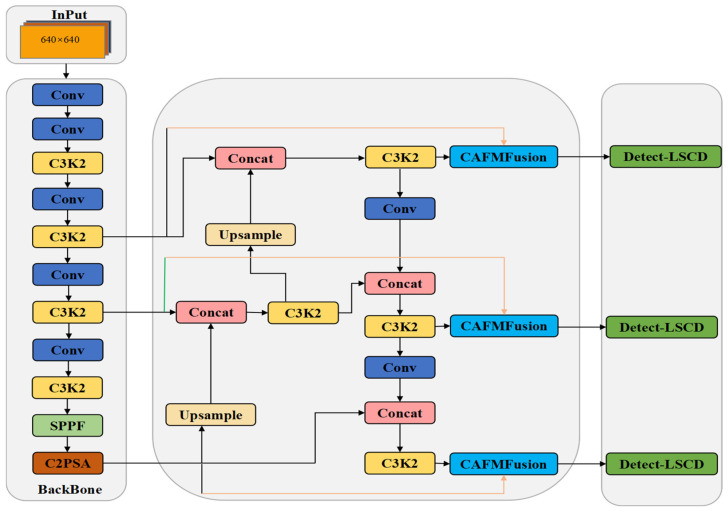
Schematic diagram of the target-detection network architecture based on the CAFM Fusion module.

**Figure 6:**
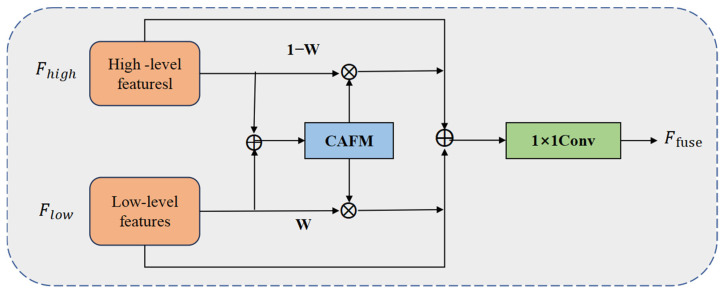
CAFM Fusion model.

The original DEA-Net model is improved by introducing the Content-Guided Attention Fusion (CGAF) mechanism, where the CGA (Content-Guided Attention) mechanism is replaced by HCANet. A lightweight C2PSA module is proposed with depthwise separable convolution and reparameterization techniques, substituting the traditional spatial attention mechanism to achieve superior local–global feature fusion. Accordingly, images captured by a monocular camera are input into the YOLOv11n model (Figure 5), and the Convolution and Attention Fusion Module (CAFM) is developed in the model, as illustrated in Figure 6. The backbone network of the model extracts features from the input images, and these are then enhanced by the CAFM Fusion module. Finally, the high-level semantic-discrimination module enables the recognition and perception of whether the vehicle accurately enters the designated area and completes the charging connection.

The proposed CAFM module is composed of a local branch and a global branch, which are designed to model local neighborhood structures and long-range contextual dependencies, respectively. The local branch extracts neighborhood information through 1 × 1 convolution, channel shuffle, and 3D convolution, with its feature output expressed in Equation (15), as follows:

(15) $$F_{\mathrm{conv}}=W_{3\times 3\times 3}(CS(W_{1\times 1}(Y)))$$

where $W_{1\times 1}$ and $W_{3\times 3\times 3}$ represent the 1 × 1 convolution and 3 × 3 convolution operations respectively, CS is the channel-shuffling operation, and Y is the input feature.

The global branch employs a self-attention-based modeling approach to capture long-range dependencies through the weighted relationships among Query (Q), Key (K), and Value (V). Its feature outputs are given by Equations (16) and (17), as follows:

(16) $$F_{\mathrm{att}}=W_{1\times 1}(\mathrm{Attention}(Q,K,V))+Y$$

(17) $$\mathrm{Attention}(Q,K,V)=V\times \mathrm{Softmax}(\frac{K^{T}Q}{\alpha })$$

where *α* is a learnable scaling factor that is used to control the distribution scale of the attention map.

The final output is obtained by fusing the local and global features, as shown in Equation (18), below:

(18) $$F_{\mathrm{out}}=F_{\mathrm{conv}}+F_{\mathrm{attention}}$$

**Figure 7:**
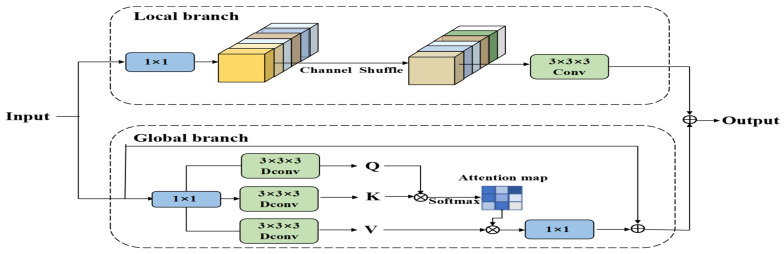
CAFM module.

The improved module is embedded into the high-level feature fusion part of the YOLOv11n detection head to enhance the model’s perception capability and feature representation for key target regions. Compared with the original structure, the CAFM module effectively integrates local details and global contextual information while maintaining light weight, demonstrating significant improvements in recognition and detection of small objects in complex scenes. The architecture of the CAFM module is illustrated in Figure 7.

### 4.2. Mosaic Data Augmentation

**Figure 8:**
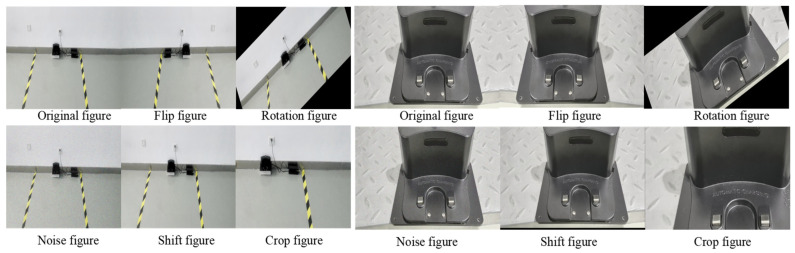
Enhancement of image effects.

In this experiment, the training data for the YOLOv11n network model were derived from a self-constructed dataset. For the collected video data, inter-frame sampling was performed by extracting one frame every 30 frames. To satisfy the requirements of deep learning for large-scale training samples and enhance the model’s generalization ability, multiple data-augmentation strategies were adopted, including random cropping, flipping, translation, noise addition, mirroring, and Mosaic augmentation. Through integration of frame sampling with data-augmentation methods, a dataset containing 2152 effective images was finally established. The images were annotated using the MakeSense tool, and the dataset was randomly divided into training, validation, and test sets at an 8:1:1 ratio. Schematic diagrams of partial augmentation results are shown in Figure 8.

### 4.3. Testing for Identification of Charging Area and Charging Pile

The model improvements and experiments in this study are all conducted on the computer configuration listed in Table 2. Despite differences in model architectures, consistent parameter settings are maintained across all experiments. The training process spans 100 epochs, processing 16 images per batch with a resolution of 640 × 640 pixels. To enhance computational efficiency, GPU acceleration is enabled, and the number of data-loading worker threads is set to four to expedite data transfer. During optimization, the Stochastic Gradient Descent (SGD) optimizer with a momentum of 0.937 is employed to minimize the loss function. To ensure reproducibility, a fixed random seed of 0 is used for all experiments.

As shown in Table 3, this paper conducts structural optimization based on YOLOv11n and compares the detection performance of the Optimized YOLOv11n with multiple mainstream lightweight object-detection algorithms (YOLOv5n, YOLOv8n, YOLOv11n) for three key target categories (cathode sheet, anode sheet, and infrared emission port). Experimental results demonstrate that the Optimized YOLOv11n proposed in this paper outperforms all counterparts in all evaluation metrics, achieving a precision (P) of 84.0% and a recall (R) of 78.1% and improving mAP@0.5 and mAP@0.5:0.95 to 78.6% and 49.7%, respectively. Compared with the original YOLOv11n model, the proposed method improves mAP@0.5 by 2.0% and mAP@0.5:0.95 by 1.0% while maintaining similar recall rates. This result fully validates the advantages and effectiveness of the proposed improvement strategy in enhancing the accuracy of detection of small targets, especially under high IoU thresholds (i.e., under stricter matching conditions).

As shown in Table 4, this paper proposes structural improvements based on YOLOv11n and conducts comparative experiments with mainstream lightweight object detection models (YOLOv5, YOLOv8n, and the original YOLOv11n) on the detection tasks of charging areas and charging piles. The results show that the improved method achieves optimal performance in both precision (P) and mAP@0.5, reaching 89.7% and 86.3%, respectively. It also achieves 60.5% in mAP@0.5:0.95, representing a 1.9% improvement compared to the original YOLOv11n. Although the recall (R) is slightly lower than that of YOLOv5 (85.2% vs. 86.1%), the overall metrics demonstrate more balanced performance. Notably, the improvement in mAP metrics better reflects the model’s comprehensive detection capabilities in terms of localization and classification. These experimental results fully validate the effectiveness and adaptability of the proposed optimization strategy based on YOLOv11n for the identification of charging areas and charging piles.

**Figure 9:**
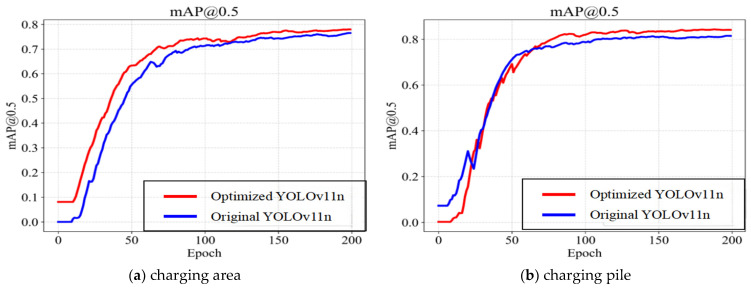
Accuracy of target-object detection.

Figure 9 compares the performance metrics for recognition of the charging area and charging pile, in which both models exhibit monotonically increasing mAP@50. The optimized YOLOv11n outperforms the original YOLOv11n: as shown in Figure 9a, for larger targets, it surpasses the original model’s mAP@50 from the start, showing faster convergence and higher precision; as shown in Figure 9b, despite initial lower precision than the original YOLOv11n, after 60 epochs, it achieves significantly better precision and convergence with a more substantial gain in accuracy, indicating superior performance in small-target detection.

**Figure 10:**
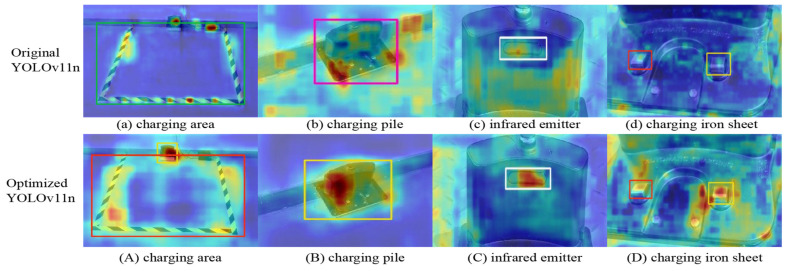
Heatmaps.

To visually demonstrate the effect of algorithm optimization, Class Activation Map (CAM) technology is introduced to generate heatmaps highlighting the salient features of specific categories in images. These heatmaps intuitively reflect the attention regions of convolutional neural networks during target feature extraction and effectively compare the feature-learning capabilities of different models. By visualizing network weights, the heatmaps highlight the regions with the strongest neural network responses, where darker colors indicate higher importance, enabling more accurate focus on target areas. Figure 10 shows the performance of the YOLOv11n model and that of the improved model in object-detection tasks as a comparative heatmap, where Figure 10a–d present the visualization results of the original YOLOv11n model and Figure 10A–D show the corresponding heatmaps of the improved model. Experimental results demonstrate that the improved model exhibits stronger responsiveness and discriminative power in feature extraction for targets of different scales, effectively enhancing the overall detection performance.

**Figure 11:**
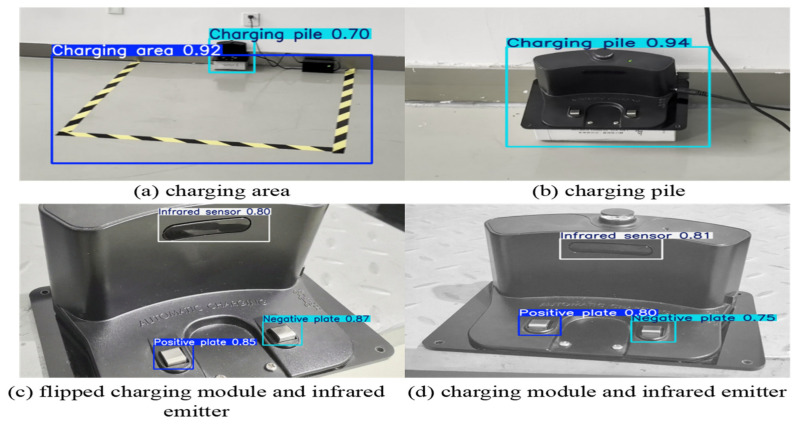
Target-recognition accuracy.

Figure 11 presents the results used to evaluate the object-detection confidence of the model in real-world scenarios. The charging area in Figure 11a is detected with a confidence of 0.92; the charging pile in Figure 11b is detected with a confidence of 0.94; and the positive/negative poles and infrared emission ports in Figure 11c are detected with confidences of 0.85, 0.87, and 0.8, respectively. Notably, the confidences for the charging area and charging pile alone both exceed the high threshold of 0.9, while the local recognition of charging piles remains above 0.8. These results indicate that the model demonstrates extremely high reliability in detection outcomes, enabling the visual recognition and localization of both the entire area and the charging piles.

## 5. Testing and Validation Based on SLAM Method

The feasibility of navigation planning for autonomous charging is validated using a ROS-based intelligent-vehicle system. The vehicle is equipped with ROS1, a front-mounted sc-mini LiDAR, rear-mounted ultrasonic radars, a ZED camera, and IMU-based chassis control. The test procedure involves scanning static maps via SLAM [20] and dynamically planning paths to charging areas through algorithms.

**Figure 12:**
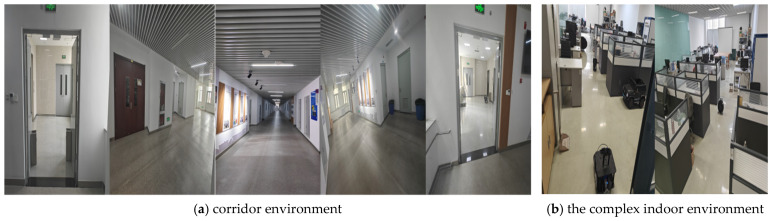
Real-world environment images.

**Figure 13:**
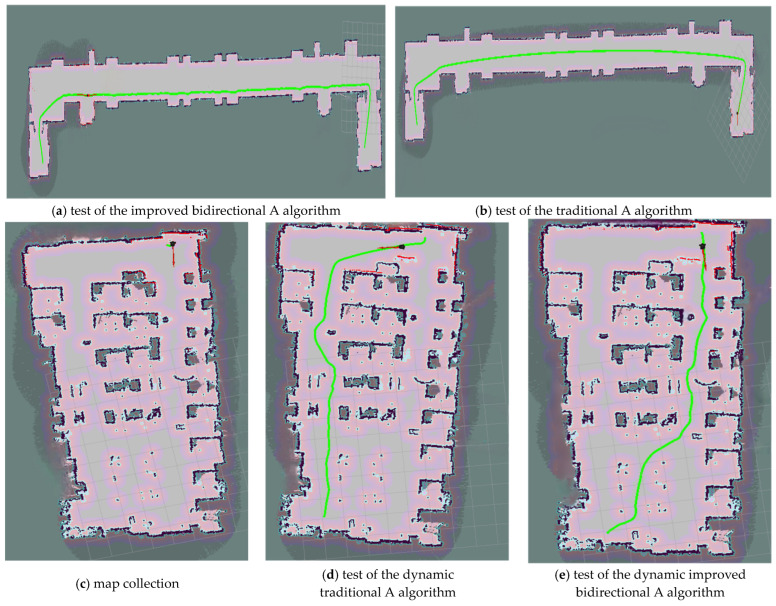
Visualization of path planning.

The long corridor (Figure 12a) measures 65 m in length and 15 m in width, while the complex indoor environment (Figure 12b) is 15.5 m long and 7.9 m wide. Charging areas with installed charging piles are set up in real-world scenarios to simulate charging-station environments. The intelligent vehicle is tested from the start point to the end point in both scenarios at a linear velocity of 0.6 m/s and an angular velocity of 0.5 rad/s. Specifically, Figure 12a shows static map-collection testing, whereas Figure 12b shows the simulation of dynamic environments via the introduction of remote-controlled carts. Figure 13a,b present results of static testing, while Figure 13c depicts indoor environment collection under static conditions. Figure 13d illustrates dynamic testing of the improved A* algorithm, and Figure 13e shows dynamic testing of the improved bidirectional A* algorithm.

Based on the results of the above tests, as shown in Table 5. The improved bidirectional A* algorithm reduces the travel distance by 7.79 m and travel time by 7.11 s compared to the improved A* algorithm in the long-corridor environment, achieving a 74.04% reduction in path-planning time and an 8.92% decrease in travel distance. In the complex indoor environment, it shortens the travel distance by 1.01 m and travel time by 1.44 s, with an 86.18% reduction in path-planning time. As visualized in Figure 13, the improved bidirectional A* algorithm demonstrates superior path-planning performance in both narrow long maps and narrow maps. Specifically, as can be seen in a comparison of Figure 13d (traditional algorithm) and Figure 13e (improved algorithm), the latter yields shorter, straighter paths with lower tortuosity. This experiment validates the algorithm’s rationality, effectiveness, and robustness across static/dynamic environments and symmetric/asymmetric maps, as evidenced by quantifiable performance metrics and visual path comparisons.

## 6. Conclusions

An improved bidirectional A* algorithm and an enhanced YOLOv11n object-recognition model are proposed in this paper and applied to a navigation system for autonomous charging of intelligent vehicles. The distance between search points is shortened by the algorithm through a greedy strategy, and redundant points are removed via a pruning strategy, thus reducing the length of the planned path. The heuristic function is redesigned by combining the number of algorithm iterations and the distance between the current point and the target point, accelerating the search speed and reducing the computation time. The path planned by the algorithm is optimized using a third-order Bézier curve to generate a smooth and continuous trajectory, enabling the vehicle to reach the destination along a smooth path. Additionally, the HCANet module is incorporated into the YOLOv11n model, and the proposed CAFM model achieves superior fusion of local and global features, enhancing the performance of small-object detection. Thus, the system integrates the optimized bidirectional A* algorithm and the improved YOLOv11n system, achieving the goal of navigation for autonomous charging.

## Figures and Tables

**Table 1:** Comparative analysis of algorithm performance.

Map	20 × 20 Mesh		50 × 50 Mesh		100 × 100 Mesh	
Algorithm	Traditional A*	Bidirectional A*	Traditional A*	Bidirectional A*	Traditional A*	Bidirectional A*
Time(s)	0.12	0.03	2.78	0.93	17.04	3.46
Number of search nodes	544	424	6592	2711	18310	10327
The Original Path (m)	28.97	28.14	71.60	70.43	136.65	135.24
The Greedy Optimized Path (m)	26.70	26.61	67.16	66.86	129.81	128.20
The Cubic Bézier Optimized Path (m)	26.86	26.73	67.57	67.20	131.37	128.89
The Smoothness of the Cubic Bézier Optimized Path	15.25	21.87	48.50	66.92	78.18	107.53

**Table 2:** Experimental environment configuration.

Category	Environmental Conditions
CPU	Inter Core i9-9900KF
Graphics	NVIDIA RTX 3080
CUDA version	12.0
Python	3.9.12
torch	2.0.0
mmcv	2.2.0

**Table 3:** Comparison of self-built datasets for charging stations and charging areas.

Model	P	R	mAP@0.5	mAP@0.5:0.95
YOLOv5n	81.5%	77.2%	77.3%	47.1%
YOLOv8n	76.5%	79.7%	76.4%	44.8%
YOLOv11n	83.1%	75.7%	76.6%	48.7%
Optimized YOLOv11n	84.0%	78.1%	78.6%	49.7%

**Table 4:** Comparison of self-built datasets for cathode sheets, anode sheets, and infrared emitters.

Model	P	R	mAP@0.5	mAP@0.5:0.95
YOLOv5	88.5%	86.1%	83.3%	59.6%
YOLOv8n	87.9%	85.8%	84.1%	60.2%
YOLOv11n	88.7%	81.2%	81.8%	58.6%
Optimized YOLOv11n	89.7%	85.2%	86.3%	60.5%

**Table 5:** Comparison of algorithm test results.

Map	Algorithm	Path-Planning Time (s)	Actual Walking Time (s)	Path Length (m)
Long Corridor	Improved A* algorithm	1.31	94.45	87.26
	Improved Bidirectional A* Algorithm	0.34	87.34	79.47
Complex Indoor Environment	Improved A* algorithm	0.94	31.22	17.04
	Improved Bidirectional A* Algorithm	0.13	29.78	16.03

## Data Availability

The raw data supporting the conclusions of this article will be made available by the authors on request.

## Abbreviations

The following abbreviations are used in this manuscript:

ACS	Automated Charging System
ANS	Automated Navigation System
ACNS	Navigation system for autonomous charging
RRT	Rapidly-exploring Random Trees
APF	Artificial Potential Field
CGA	Content-Guided Attention
CAFM	Convolution and Attention Fusion Module
CAM	Class Activation Map
